# Meeting Reports

**DOI:** 10.1155/S1463924679000638

**Published:** 1979


### Meeting Reports

### Third European Congress of Clinical Chemistry

The Congress was held from the 4-8 June 1979 at the Metropole Hotel, Brighton, UK. After an introductory speech by Professor T.P. Whitehead and a welcome from Professor B.E. Clayton for the Association of Clinical Biochemists. Dr. R. Dybkaer, President of the International Federation of Clinical Chemistry, then officially opened the Congress. The Congress was organised to provide a good mix of scientific communication in the form of Plenary lectures by distinguished scientists, Symposia on selected topics, Seminars by industrial contributors and Contributed Paper sessions by scientists with a wide range of interests. The latter three groups generally ran in parallel with each other and with Poster sessions covering a wide range of topics. Throughout the period of the Congress a massive exhibition of clinical chemistry instrumentation was held on two floors. A fine social programme included a formal banquet and an outing to the 200th Derby. Whilst the objective of this report is specifically related to automation it is necessary to note the plenary lectures.

Astrup [1] in his plenary lecture drew attention to both the criticisms and advantages of modern trends in clinical chemistry. Porter [2] gave a fine plenary lecture in which he showed the way x-ray crystallographic studies are elucidating the mechanism of antibody/antigen binding and the structure, assembly and mode of activiation of the components of complement. A highlight of the Congress was the superb lecture given by Galjaard [3].Advances now make it possible to diagnose many metabolic disorders by genetic counselling prior to conception, tests on cells and cord blood in utero and on patients either at risk or with overt symptoms.

Marks [4] discussed the rapidly growing area of drug monitoring in which clinical chemistry is more concerned with treatment than diagnosis. Thore [5] in his plenary lecture gave an excellent account of the emerging science of luminescence in clinical chemistry.

Lever [6] in a controversial plenary lecture outlined the criteria for classifying hypertension and then presented evidence to cast doubt on the sub-division of essential hypertension into low-renin and normo-renin forms. Granstrom [7] gave a lucid account of the start of the art in prostaglandin and thromboxane research. Lever [8] in discussing renal failure drew attention to the isolation of a chromatographic fraction of M W 1000 – 1500 with the ability to inhibit haemoglobin formation.

The International Federation of Clinical Chemistry organised a symposium on 'Decision Criteria for Selecting Analytical Instruments' in which members of the Expert Panel on Instrumentation detailed the ways in which more informed decisions on the choice of costly automatic equipment can be made, to suit the needs of each laboratory. A starting point is to encourage manufacturers to provide the right information; the use of cost/benefit studies was considered important; the value of sound evaluations and contact with colleagues with previous experience of intended purchase was emphasised. Finally education of laboratory managers to consider the effects on laboratory organisation was underlined and in this, computer simulation studies are proving valuable. Papers in this session will be published in the Journal of Automatic Chemistry 1979 October issue.

A symposium on techniques in data handling included a contribution by Lave [9] on the relativements of bar coding and other techniques. The size of each code can be a problem and the desirability of having human readable code additionally, was emphasised. Knill-Jones [10] discussed clinical aspects of data reduction and an excellent contribution by Healy [11] highlighted the use and abuse of statistics in clinical chemistry. Hall [12] produced some interesting thoughts on the use of computers to find trends in health and disease over a period of time at the individual level and so circumvent the vagaries of the normal range.

A symposium on approaches in clinical chemistry analytical techniques included an account of an exciting development in 'big science' by Anderson [13]. He revealed the ability to quantitate and detect, in principle, tens of thousands of proteins in biological samples by 2-D isoelectric focussing and acrylamide gel electrophoresis. Techniques borrowed from astronomy and space science were used to position and quantitate spots formed by dye or radio location techniques. It is hoped that by building a library of data intrusion into clinical chemistry will be steady. Przybylowicz [14] presented an account of the new multilayer film technology developed by Eastman Kodak. The instrumentation was also discussed and the advantages given to the user by simplicity standardisation and freedom from reagent storage problems were emphasised.

Other symposia focussed on the assessment of the efficiency of clinical biochemical tests and on standardisation in clinical chemistry. These areas are clearly of considerable importance judging by the number of contributed papers and posters presented on these subjects. A large number of Contributed Papers sessions ran throughout the Congress and two poster sessions were held daily, and again areas of current interest were highlighted. A total of Seven Industrial Seminars were held. A presentation of the past, present and future of centrifugal analysis was presented by IL [15] including a contribution by the inventor (Dr. Anderson). The importance of the technique in minimising the effects of instrumental drift by rapid sequential spectroscopy was emphasised and attention was drawn to future developments in the form of UV transparent rotors, fluorescence measurement and techniques for ESR, blood grouping and coagulation measurement.

Eastman Kodak [16] presented their multilayer film technology emphasising the care which has been taken to arrive at sensible precision and accuracy goals for each analyte and a demonstration of how these goals have been met in external trade tests in the USA and in Europe. An indication of the scope of the technology was hinted at by Dr. E. Przybylowicz who stated that ion-selective electrode, fluorescence, immunoassay and NAD/NADH based systems were under development.

Du Pont [17] introduced the ion-specific electrode attachment for the ACA analyser and their prep 1 - HPLC sample preparation and analysis system. The latter device should prove an invaluable aid in toxicology where a number of samples must be extracted under carefully controlled conditions prior to analysis by HPLC.

Other industrial seminars were by Technicon[18] who introduced the 'STAR' continuous radioimmunoassay analyser, Syva [19] who described the EMIT technique, and LKB Clinicon [20] who presented the 'PRISMA', their programmable selective modular analyser.

A most impressive exhibition of clinical chemistry equipment was mounted on two floors. Exhibits ranged from the smallest items to aid the manual laboratory worker to comprehensive multi-channel analysers with on-line data processing systems. In addition, many books and journals were on view.

Undoubtedly the Third European Congress of Clinical Chemsitry was an outstanding success and served to stimulate great interest and exchange of views in all the important areas of clinical chemstiry. The organisation was excellent and yet never obtrusive and all those who took part are to be warmly congratulated.

*Mark S. Stoll*

BIBLIOGRAPHY
[1] *Current trends in Clinical Chemistry,* P. Astrup. Professor of Clinical Chemistry, University of Copenhagen, Denmark.
[2] *The Molecular Basis of Immunity,* R.R. Porter. Whitely Professor of Biochemistry, University of Oxford, UK.
[3] *Early Diagnosis and prevention of congenital diseases,* H. Galjaard, Department of Cell Biology and Genetics, Erasmus University, Rotterdam, Netherlands.
[4] *The role of drug monitoring in clinical chemistry,* V. Marks. Professor of Clinical Biochemistry, University of Surrey, Guildford, UK.
[5] *Luminescence in clinical analysis,* A. Thore. Senior Research Officer, Head of the Sector for Bacteriology, Swedish National Defence Research Institute, Sundyberg, Sweden.
[6] *The clinical chemistry of hypertension,* A.F. Lever. Director Medical Research Council Blood Pressure Unit, Western Infirmary, Glasgow, UK.
[7] *Aspects on measurements of prostglandins and thromboxanes.* E. Granstrom, Assistant Professor in Medical and Physiological Chemistry, Karolinska Institute, Stockholm, Sweden.
[8] *Middle molecules in renal failure,* H.H. Leber. Medical University Clinic, Giessen, Federal Republic of Germany.
[9] *Data entry by bar coding and other techniques,* D. Laue (Cologne, FRG).
[10] *Clinical aspects of data reduction,* R. Knill-Jones (Glasgow UK).
[11] *Statistical approaches to data reduction,* M.J.R. Healy (London UK).
[12] *Problem-orientated records and health indicators,* P. Hall (Stockholm, Sweden).
[13] *Applications of two-dimensional protein gene mapping in clinical chemistry,* N.G. Anderson (Argonne, Ill. U.S.A).
[14] *Ektachem Technology – a new method for quantitative determinations in clinical chemistry* (Rochester, NY, USA).
[15] Instrumentation Laboratory (UK) Ltd, Kelvin Close, Birchwood Science Park, Risley, Warrington, Cheshire, WA3 7PB.
[16] Kodak Ltd., Victoria Road, South Ruislip, Middlesex.
[17] Du Pont (UK) Ltd, 64 Wilbury Way, Hitchin, Hertfordshire SG4 OUR.
[18] Technicon Instruments Co. Ltd, Hamilton Close, Basingstoke, Hants.
[19] Syva, St. Ives House, St. Ives Road, Maidenhead, Berks.
[20] LKB Clinicon Systems Ltd, Bell Lane, Lewes, East Sussex, BN7 1LG.

### Automation in Clinical Chemistry

The first international Symposium on Automation in Clinical Chemistry was held in the Ciudad Sanitaria de la Seguridad Social Principes de Espana, Barcelona, Spain, from March 7-10 1979.

The aim of this first meeting in Spain was to provide an international forum for the discussion of this important subject in the context of conditions in Spain and elsewhere. The presentations and round-table conferences covered three aspects of the subject: (i) automation as an end in itself, including the current developments in instrumentation and computerisation, especially with respect to enzyme and lipid measurements; (ii) quality control materials and their application and use for inter- and intra-laboratory quality control; (iii) practical examples of the impact of the introduction of automation. The last day of the meeting was devoted to the problems of automation in the private laboratory. Comprehensive reports of all papers and round table conferences will be published in: Dto. Analisis Clinicos, C.S.S.S. Principes de España, Barcelona.

In his opening remarks, the President of the Symposium, Dr. Fernández Simó, Jefe Dto. de Analisis Clinicos, expressed the hope that all present would speak freely in the lengthy discussion periods which had been arranged. He introduced the main speakers who gave papers as follows:

Dr. F.L. Mitchell (Division of Clinical Chemistry, Clinical Research Centre, Harrow, UK) dealt with the recent advances in the automation of clinical chemistry, the progress which has been achieved since the introduction of continuous flow analysis over 20 years ago, and the difficulties now facing users because of the considerable choice of instrumentation currently available from the new and large industry which has evolved. He drew attention to the problem of choice for the large laboratory between a single multichannel analyser, and the new small highly versatile stand alone analysers linked by computer. The versatility and low price of microprocessors makes it possible to apply computer control and processing where economic considerations were previously prohibitive. The impact that the introduction of layer chemistry will have on almost all types of laboratory was also discussed.

Dr. R. Jensen (Biologiste des Hôpitaux, Hôpital Saint-Aindré. C.S. Bordeaux) covered the difficulties in directing the clinical laboratory problems relating to increasing work and data throughout, and the more stringent controls which are now required were discussed. He drew attention to four stages of development which have occurred in his own laboratory over the last 25 years: (i) no automation as currently understood; (ii) the installation of single channel analysers allowing the daily output of analyses to increase from 250 to 450; (iii) the use of Technicon SMA instruments with limited computer and data processing links between the laboratory and other departments; (iv) the installation of the Technicon SMAC. The changes necessitated an increase in the skills required of the laboratory directors. In addition to the knowledge in medicine and chemistry gained at university, he must now exercise skills in architecture, electronics, computer programming, management, psychology, etc. Many of these qualities are also required in technical staff whose selection and training pose increasing problems.

Dr. A. Corominas (Servicio Análisis Clinicos, R.S.S.S., Barcelona, Spain) reviewed the automated methods and systems available for lipid determinations. Selection of the most appropriate measurement instrument or system to be used, depends upon the number of samples, the type of patient and whether preventive medicine or diagnosis and treatment are received. He enumerated the manual methods used for the determination of total lipids, lipoproteins and their components, cholesterol, phospholipids and fatty acids, particularly concentrating on the variety of methods currently applied to cholesterol assay-fluorimetric, colorimetric, gravimetric, chromatographic, electrochemical and enzymatic. The enzymatic methods for triglyceride determination were mentioned together with titrimetric, colorimetric, fluorimetric and radiochemical methods for the measurement of fatty acids. Finally he discussed the use of lyophilised sera in quality control.

Dr. J.P. Colombo (Chemisches Zentrallabor-Inselspital, Bern, Switzerland) confined his discussion to the interaction between a laboratory computer system and the hospital information system. Though the functions of both may be carried out on a centralised data processor, they must be developed separately, and introduction of the information system should be completed in stages depending upon the various medical and clerical units to be served in the hospital. In the system described by Dr. Colombo, each unit can access information depending upon the requirements of the unit concerned. In its laboratory management role, the computer receives information from instruments, on- and off-line. This allows the separate development of individual instruments. By keeping the hospital information and laboratory management roles separate within the single processor, the autonomy of the laboratory is maintained while allowing the maximum availability of laboratory information within the hospital.

Dr. C. Collombel (Biologiste des Hopitaux, Hopital des Charpennes, Lyon, France) dealt with the problems involved in the application of automation to the measurement of enzyme activity. Requirements for this are currently increasing between 20 and 30% per year. The methods of choice are kinetic and require special attention; automation is necessary if the reliability and reproducibility of results are to be achieved and maintained.

In the choice of systems it is important to take into account the parameters recommended by governmental or international organisations such as the International Federation for Clinical Chemistry and the World Health Organisation. The important areas to consider are – temperature, dilution of enzyme, any pre-incubation period, method of reaction initiation, the method of result calculation from raw data and the overall time to be allowed for the kinetic measurement. Based on a comprehensive survey of kinetic analysers currently available in France, he reviewed the technical features which influence the performance of the instrument.

Dr. T.P. Whitehead (Department of Clinical Chemistry, Queen Elizabeth Medical Centre, Birmingham, U.K.) who organises the United Kingdom Quality Control Scheme, explained the evolution of the Scheme over the last decade. He showed the improvement in the quality of laboratory output which had ensued. The Scheme indicates the performance of different methods used for individual types of analysis and this feature has had a substantial influence upon the choice of method by the participants. Many foreign laboratories now take part in the British Scheme. The different patterns of control, displayed in other countries is noteworthy.

Dr. L. Patiño (Warner Lambert International, PO Box 377 Morris Plains, New Jersey, 07950, USA) dealt with the use of lyophilised serum for quality control in the automated laboratory. Often large commercial laboratories producing quality control materials can obtain so-called 'standard' material by averaging results obtained in many reference laboratories. Another method of obtaining analytical values for a batch of material is to average the results obtained in a quality control scheme with many participating laboratories. He explained the complexity of the processes used to obtain the large batches of sera required, and indicated in detail the manufacturing procedures designed to produce the necessary characteristics of a batch control material.

Dr. A. Funes (Cerba International, Maffliers. 95560 Montsoult, France) discussed the problems of automating the more esoteric assays such as those for the measurement of steroids and various drugs in blood and urine. In most cases only semi-automation is possible, but this is often extremely valuable, for example, in the extraction of urine. He described automation used in his laboratory for various stages in a number of difficult assays. It is usually necessary to design a system specifically for a certain laboratory. The various stages can be connected by microprocessor control which can also monitor quality at selected points in the system.

Four round-table discussions were organised with some seven or eight experts participating in each particular field. All members of the Symposium joined the discussions and lively exchanges of views took place.

Dr. F.L. Mitchell presided over the discussion problems involved in the automation of a large hospital laboratory. Problems discussed were supply of instruments for normal routine measurements and the special requirements for emergency and paediatric work. The cost-effectiveness of large expensive machines was considered in comparison to the efficient operation of small versatile units with a lower initial capital cost. The merits of centralisation were discussed in relation to overall operational efficiency.

Dr. R. Galimany (Hospitalet de Llobregat, Barcelona) explained the present situation in Spanish laboratories where, because of a lack of information, automation had not progressed as rapidly as elsewhere.

Lastly, on request, Dr. Mitchell detailed the work of the Expert Panel on Instrumentation of the International Federation for Clinical Chemistry. He outlined papers to be published shortly which contained suggestions to be used by manufacturers in describing their instruments and their characteristics.

Dr. C. Collombel presided over the discussion of the automation of the laboratory using non-computerised systems. The first discourse concerned the presentation of the case for laboratory automation to a hospital's management. The points covered included consideration of the benefits for the society for which the laboratory functions, the hospital organisation, the medical staff and finally the staff of the laboratory itself. It was agreed that above a certain workload, automation and computerisation must be as complete as possible, dealing with all procedures from sample collection to the delivery of results.

Opinions differed as to the degree to which emergency samples should be integrated into a routine load. It was agreed that computerisation was beneficial only if considered for its value in correlating results with the clinical data from a patient. In discussing staff education, opinions differed as to whether analysers should be operated by specially trained technicians, or whether the laboratory staff should be able to operate them.

Dr. G. Siest presided over the discussion of the application of quality control to the automated laboratory. The discussants first dealt with the errors originating before a specimen was analysed. These can be most important and are difficult, if not impossible to control. Problems were considered arising from the stability of control materials during their delivery from manufacturer to laboratory. The relative merits of human and animal preparations were discussed together with the problems produced by lyophilisation and reconstitution of specimens.

The importance and relative merits of both internal and external control were stressed, particularly the frequency of inserting specimens for internal quality control. The possibilities of setting up a national quality control scheme for Spain were also discussed. Professor Whitehead invited all members of the Symposium to take part in the United Kingdom Quality Control Scheme for a trial period.

T. Brugerie (Cerba International, Maffliers, 95560 Montsoult, France) presided over the discussions on automation for a private independent laboratory. Each member of the round-table dealt with a specific aspect of the functioning of an independent laboratory; the technical difficulties experienced in the operation of many independent laboratories were exposed and the profitability of automation as compared with manual operation was also discussed.

A recent survey by French clinical chemists had shown that the productivity per laboratory worker does not increase once a certain workload has been attained. This could be due mainly to an increasing proportion of specialized tests which are difficult to automate.

For the introduction of automation into a general pathology laboratory it is important to consider separately the specialities biochemistry, immunology, toxicology, microbiology and haematology and for each to study the numbers of samples to be processed; the precision and accuracy required, and the overall time which can be allowed for the production of results. Computerization of data processing is extremely cost effective especially when on-line operation is used. Renting equipment needs careful consideration especially in relation to the cost for each analysis and the relationship between total cost and the volume of workload.

In an independent laboratory emergency work includes a wide range of analyses since it is difficult to control the selection of tests. Because of this automation needs to be arranged so that it covers both the emergency and routine load.

For the rural laboratory screening a population of about 10,000, the first tests to be considered for automation are for haematology – erythrocyte and leucocyte count and haemoglobin and haematocrit measurement. Though glucose is more commonly requested, the four haematological tests are more difficult to complete. The more common biochemical determinations are best done using semi-automation dispensors, diluters, etc.

*R. Galimany and F.L. Mitchell*

### Analytical Quality Control

This two day meeting, held at Stirling University, Scotland, on 7-8 June 1979, was a worthy successor to the meeting held last year at St Andrews when the Automatic Methods Group (Analytical Division, Chemical Society) also collaborated with the Scottish Region and others in the planning and organisation. An attendance of over 160 delegates fully justified the hard work put in by local members of the Scottish Region Committee.

The meeting opened with a plenary address by C.A. Johnson, of the British Pharmacopoeia Commission, in which he gave a characteristically witty and discursive survey of the present state of quality control. He commented unfavourably on the unneccessarily demanding specifications in step with improvements in instrumentation and analytical techniques. Professor A.R. Rogers examined the use made by analysts of statistics, commenting particularly on the fact that although they usually received some training in elementary statistical techniques only rarely were they able to correctly interpret the results of applying them. J.D. Chamberlain gave an exposition of Cumulative Sum Techniques (CUSUMS) and demonstrated their advantages over other control chart techniques. In a survey of methods of acceptance sampling R. Caulcutt commented unfavourably on the comprehensibility of BS6001 which was written with the use of technical terms that limited its utility for analytical chemists and quality controllers.

T.E.V. Horsley reflected on more than 40 years of quality control in the pharmaceutical industry and commented, not altogether favourably, on the increasing complexity of modern life. He reviewed the many advantages of automation in pharmaceutical quality control laboratories but said that experience had taught him that although automation was essential, one must not lose sight of the valuable specialised capabilities of the human senses, particularly the eyes and hands working in conjunction with the brain.

Dr. D. Betteridge surveyed recent developments in the field of microprocessors with particular emphasis on their advantages and limitations. He illustrated how microprocessors allowed analytical chemists to adopt new approaches to their problems by describing his involvement in the development of an automated titrator. He concluded by describing some of the uses that could be made of microcomputers such as the PET.

D.E. Faulkner presented a paper that reveiwed the application of AutoAnalyzer techniques to the work of the Wellcome Foundation, paying particular attention to hardware and alternative methods of data handling.

G.K.E. Copeland described the analytical techniques used at the Laboratory of the Government Chemist to produce the cigarette tar and nicotine content league tables that are published twice a year by the UK Departments of Health. A fully automated system measures the tar, nicotine, water and carbon monoxide levels produced by smoking cigarettes on an automatic machine. Data handling is carried out on a central computer with instruments linked on-line, this facilitating the use of analytical quality control techniques.

The second day opened with a plenary lecture from R. Sawyer, Superintendent of the Food and Nutrition Division at the Laboratory of the Government Chemist, who explained the aims of EEC legislation and the work being carried out to ensure harmonistion. He described the differing enforcement methods used in the various Common Market countries and pointed out that the ability of different countries to put their own interpretation on Directives was a valuable factor in ensuring their acceptance, although this could lead to subsequent problems for the analyst. The United Kingdom differed from other Common Market countries who generally favoured the adoption of standard analytical methods – an approach that had not been favoured in UK legislation in the past. Mr. Sawyer concluded an interesting, and often amusing, lecture with some personal thoughts on the need for the quality control of EEC legislation.

The three following papers from S.J. Anderson, S.V. Ayers and J.E. Pentelow surveyed the use of quality control in the beer, wine and plastic packaging industries respectively.

Professor G. Ghersini, from Milan, described some novel control charts that he had developed to assist the monitoring of radionuclide levels in the electrical power generating industry. He was followed by T.C. Foster who described the problems of making consistent additions to animal feedstuffs formulations. The meeting concluded with a paper from G.A. Best who gave an account of analytical quality control methods used in the laboratories of the Clyde River Purification Board and the workings of the Harmonised Monitoring Scheme.

Advantage was taken of the proximity to Gleneagles to round off the meeting by holding the second Technicon Golf Tournament at the conclusion of the scientific programme. A dozen golfers accompanied by about sixteen enthusiastic spectators spent an enjoyable few hours on the 18 holes of the King's Course. During the ensuing dinner at Blairlogie House Hotel the prizes were presented by David Thomson on behalf of the Technicon Instrument Co. Ltd. The first prize, and the handsome Technicon Golf Trophy, went to Dr. B.J. Woodhall of ICI Pharmaceutical Division, the second prize to Mr. D.J. Evans of Smith, Kline and French and the third prize to Dr. A.G. Davidson of Strathclyde University. The hotly contested booby prize went to R.N. Thornhill of Smith, Kline and French.

*D. Porter*

